# Effectiveness of negative pressure wound therapy versus conventional dressings in emergency laparotomy: a systematic review and meta-analysis of randomized controlled trials

**DOI:** 10.1590/0102-672020260000015e1944

**Published:** 2026-07-20

**Authors:** Francielle de Souza ANTONINI, Maria Eduarda Caetano Batista de PAIVA, Marcílio de OLIVEIRA, Fabricio Inacio de Moraes NASCIMENTO, Rodrigo Ormanes MASSOUD, Henrique Baggio PIVETTA, Leticia Gatti FONSECA, José Victor Gomes COSTA, Yekaterina ZELENSKAYA, Zarpana AKBARI, Maria Isabel Lopez DUQUE, José Eduardo de AGUILAR-NASCIMENTO, Gunther Peres Pimenta

**Affiliations:** 1 Hospital Regional de São José Doutor Homero de Miranda Gomes – São José (SC), Brazil.; 2 Hospital Heliópolis Hospital – São Paulo (SP), Brazil.; 3 Faculdade de Medicina de Barbacena – Barbacena (MG), Brazil.; 4 Hospital Nove de Julho – São Paulo (SP), Brazil.; 5 Universidade do Estado do Pará – Belém (PA), Brazil.; 6 Universidade do Sul de Santa Catarina – Palhoça (SC), Brazil.; 7 Centro Universitário São Camilo – São Paulo (SP), Brazil.; 8 Hospital Alemão Osvaldo Cruz – São Paulo (SP), Brazil.; 9 Kazakh National Medical University – Almaty, Kazakhstan.; 10 Kabul Medical University – Kabul, Afghanistan.; 11 CES University, Department of Surgery – Medellín, Colombia.; 12 Centro Universitário de Várzea Grande – Várzea Grande (MT), Brazil.; 13 Universidade Federal do Mato Grosso – Cuiabá (MT), Brazil.

**Keywords:** Surgical Wound Infection, Negative Pressure Wound Therapy, Laparotomy, Emergencies, Infecção do Sítio Cirúrgico, Tratamento de Ferimentos com Pressão Negativa, Laparotomia, Emergências

## Abstract

**Background:**

Surgical site infections represent a significant complication after emergency laparotomy, contributing to increased morbidity, mortality, and healthcare costs. Negative pressure wound therapy (NPWT) may reduce the incidence of surgical site infections; however, evidence from randomized controlled trials remains inconsistent.

**Aims:**

To present a systematic review and meta-analysis, comparing NPWT with conventional dressings in emergency laparotomies.

**Methods:**

Systematic searches were conducted according to the Cochrane Handbook and reported according to the PRISMA 2020 guidelines. The PubMed, Embase, and Cochrane CENTRAL databases were searched since their inception, using terms related to emergency laparotomy, NPWT, and conventional dressings. The reference lists of included studies were manually examined to identify additional eligible trials. Only randomized controlled trials that compared NPWT with conventional dressings in emergency abdominal surgeries and that reported surgical site infection rates or other clinical outcomes were included. The primary outcome was the incidence of surgical site infection. Secondary outcomes included wound dehiscence, seroma formation, length of hospital stay, and 30-day mortality.

**Results:**

Eight randomized controlled trials, totaling 1,377 patients (707 in the NPWT group and 670 in the conventional dressing group), were included. NPWT significantly reduced the risk of surgical site infection (risk ratio [RR] 0.43; 95% confidence interval [CI] 0.26–0.73; p=0.002; heterogeneity [I2]=76%) and wound dehiscence (RR 0.32; 95%CI 0.18–0.58; p=0.0002). No significant differences were observed regarding seroma formation (RR 0.59; 95%CI 0.34–1.01; p=0.05), length of hospital stay (mean difference [MD] -0.39 days; 95%CI -1.15– 0.36; p=0.92) or mortality (RR 0.96; 95%CI 0.55–1.68; p=0.88).

**Conclusions:**

This systematic review and meta-analysis of randomized clinical trials suggests that NPWT reduces the incidence of surgical site infection and wound dehiscence after emergency laparotomy, supporting its use in high-risk patients.

## ARTICLE HIGHLIGHTS

Postoperative infections have a significant impact on surgical care, contributing to increased morbidity, length of hospital stay, and healthcare expenditures;

Strategies to reduce postoperative complications have increasingly focused on perioperative optimization through evidence-based, multimodal protocols;

The ACERTO guidelines on perioperative nutritional interventions in elective general surgery were developed to enhance postoperative recovery and reduce overall morbidity;

Numerous investigations have effectively furnished substantial evidence pertaining to the clinical advantages associated with negative pressure wound therapy;

This systematic review and meta-analysis of randomized clinical trials suggests that negative pressure wound therapy reduces the incidence of surgical site infection.

## Summary

**CENTRAL MESSAGE:**

Surgical site infections are a major postoperative complication after emergency laparotomies, contributing substantially to morbidity, mortality, and healthcare costs. Strategies to reduce postoperative complications have increasingly focused on perioperative optimization through evidence-based, multimodal protocols. Retrospective analyses have demonstrated the efficacy of intermittent negative pressure wound therapy in diminishing the incidence of surgical site infections following laparotomy in patients undergoing gynecological and general surgical procedures. Nonetheless, the findings derived from randomized controlled trials have yielded inconsistent results.

**PERSPECTIVES:**

This original systematic review and meta-analysis of randomized controlled trials demonstrated the efficacy of negative pressure wound therapy in reducing the incidence of surgical site infections and wound dehiscence among patients undergoing emergency laparotomy when compared to conventional dressings.

## VISUAL ABSTRACT

**Figure:**
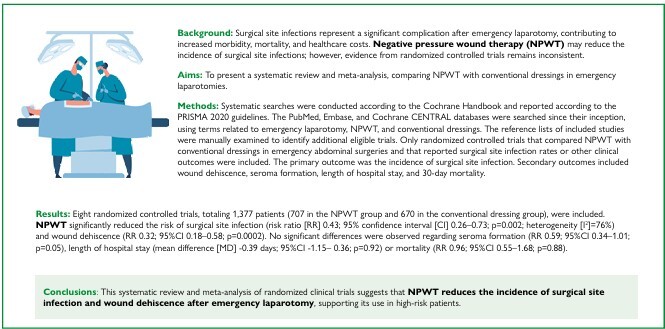

## RESUMO

**Racional:**

As infecções do sítio cirúrgico representam uma complicação relevante após laparotomia de emergência, contribuindo para o aumento da morbidade, da mortalidade e dos custos em saúde. A terapia por pressão negativa em feridas (TPNF) pode reduzir a incidência de infecções do sítio cirúrgico; entretanto, as evidências provenientes de ensaios clínicos randomizados permanecem inconsistentes.

**Objetivos:**

Apresentar uma revisão sistemática e metanálise, comparando a TPNF com curativos convencionais em laparotomias de emergência.

**Métodos:**

Foram realizadas buscas sistemáticas de acordo com o Cochrane Handbook e relatadas conforme as diretrizes PRISMA 2020. As bases PubMed, Embase e Cochrane CENTRAL foram pesquisadas desde a sua criação, utilizando termos relacionados à laparotomia de emergência, TPNF e curativos convencionais. As listas de referências dos estudos incluídos foram examinadas manualmente para a identificação de ensaios adicionais elegíveis. Foram incluídos apenas ensaios clínicos randomizados que compararam a TPNF com curativos convencionais em cirurgias abdominais de emergência e que relataram taxas de infecção do sítio cirúrgico ou outros desfechos clínicos. O desfecho primário foi a incidência de infecção do sítio cirúrgico. Os desfechos secundários incluíram deiscência da ferida, formação de seroma, tempo de internação hospitalar e mortalidade em 30 dias.

**Resultados:**

Oito ensaios clínicos randomizados, totalizando 1.377 pacientes (707 no grupo TPNF e 670 no grupo curativo convencional), foram incluídos. A TPNF reduziu significativamente o risco de infecção do sítio cirúrgico (razão de risco [RR] 0,43; intervalo de confiança [IC] 95% 0,26–0,73; p=0,002; heterogeneidade [I2]=76%) e de deiscência da ferida (RR 0,32; IC95% 0,18–0,58; p=0,0002). Não foram observadas diferenças significativas quanto à formação de seroma (RR 0,59; IC95% 0,34–1,01; p=0,05), ao tempo de internação hospitalar (diferença de médias [DM] -0,39 dias; IC95% -1,15–0,36; p=0,92) ou à mortalidade (RR 0,96; IC95% 0,55–1,68; p=0,88).

**Conclusões:**

Esta revisão sistemática e metanálise de ensaios clínicos randomizados sugere que a TPNF reduz a incidência de infecção do sítio cirúrgico e de deiscência da ferida após laparotomia de emergência, apoiando seu uso em pacientes de alto risco.

## INTRODUCTION

 Postoperative infections have a significant impact on surgical care, contributing to increased morbidity, length of hospital stay, and healthcare expenditures. Strategies to reduce postoperative complications have increasingly focused on perioperative optimization through evidence-based, multimodal protocols. The Aceleração da Recuperação Total PósOperatória (ACERTO, Acceleration of Total Postoperative Recovery) guidelines on perioperative nutritional interventions in elective general surgery were developed to enhance postoperative recovery and reduce overall morbidity, highlighting the importance of perioperative care optimization as a potential factor influencing postoperative infectious outcomes^12^. Surgical site infections (SSIs) are a major postoperative complication after emergency laparotomies, contributing substantially to morbidity, mortality, and healthcare costs^4,7,11,15,25^. Reported incidence rates range from 16.2 to 56.4%, depending on factors such as patient comorbidities, surgical techniques, and postoperative care^13,16,22,45,52,53^. The costs associated with SSIs are substantial due to increased hospital stays, readmissions, and additional medical care required for affected patients^22^. These infections not only impact patient health outcomes but also contribute to escalating healthcare expenses^6,53^. Wound dehiscence is also a critical concern, with rates of 0.3 to 3.5% in the general surgical population and up to 10% among elderly patients undergoing emergency procedures^26,34^. These figures underscore the urgent need for effective strategies to mitigate the risk of SSIs, particularly in high-risk surgical populations such as those undergoing emergency laparotomy. 

 Numerous investigations have effectively provided substantial evidence pertaining to the clinical advantages associated with negative pressure wound therapy (NPWT)^23^. Retrospective analyses have demonstrated the efficacy of intermittent negative pressure wound therapy (iNPWT) in diminishing the incidence of SSIs following laparotomy in patients undergoing gynecological and general surgical procedures^27,32,33,35,40^. Nonetheless, the findings derived from randomized controlled trials (RCTs) have yielded inconsistent results^2,8,50^. 

 Several prophylactic strategies have been proposed, including antibiotic prophylaxis, subcutaneous drainage, and NPWT^36^. NPWT applies sub-atmospheric pressure (typically 50–125 mmHg) via a sealed dressing connected to a vacuum pump and is hypothesized to enhance wound healing by improving tissue perfusion, reducing edema, and removing exudate and infectious material^12,15,24^. Evidence from orthopedic, vascular, cardiothoracic, and gynecological surgery suggests that NPWT may lower SSI rates in closed incisions^3,14,18,39,41,49,51^. 

 However, data on its effectiveness following emergency laparotomy remain inconsistent. While an earlier metaanalysis including both observational studies and RCTs found significant reductions in SSIs, wound dehiscence, and overall wound complications with NPWT, more recent large-scale RCTs have reported no significant differences compared with standard dressings^23,38,47,51^. To our knowledge, no meta-analysis on this topic has exclusively focused on RCTs. Thus, the present study synthesized RCT data to evaluate NPWT’s effect on patients undergoing emergency laparotomy. 

## METHODS

 This systematic review and meta-analysis were conducted in accordance with the Cochrane Handbook for Systematic Reviews of Interventions and were reported following the Preferred Reporting Items for Systematic Reviews and Meta-Analyses (PRISMA) 2020 statement^21,29^. The review protocol was prospectively registered in International Prospective Register of Systematic Reviews (PROSPERO, CRD 420251079600; June 24, 2025). 

### Search strategy and study selection

 PubMed, Embase, and the Cochrane CENTRAL were searched from inception to May 2025, using the terms “emergency laparotomy”, “negative wound therapy”, “NPWT”, “NPT”, “vacuum-assisted closure”, and “conventional dressings”, combined with the Boolean operators AND/OR. 

 After removal of duplicates, two authors (F.S.A. and M.C.P.) independently screened titles and abstracts of retrieved records in Rayyan software. Full texts of potentially eligible studies were then reviewed for final inclusion or exclusion. Reference lists of included studies, relevant RCTs, and previous reviews were also manually searched. Discrepancies were resolved by consensus with the senior author (G.P.P.). 

### Eligibility criteria

 Studies were included if they met the following criteria:

1. Enrolled adult patients undergoing emergency abdominal surgery;
2. Directly compared NPWT to conventional dressings;
3. Reported at least one outcome of interest; and
4. Were designed as RCTs.

Studies were excluded if they:

1. Were non-comparative;
2. Involved elective procedures; or
3. Were trials without outcomes of interest.

### Data extraction and quality assessment

 Two authors (F.S.A. and M.C.P.) independently extracted data according to predefined criteria. Extracted baseline characteristics included age, sex, body mass index, comorbidities (hypertension, diabetes, malignancy), serum albumin, smoking status, and American Society of Anesthesiologists (ASA) classification, stratified by intervention. Surgical details such as Centers for Disease Control and Prevention (CDC) wound classification, diagnosis, blood loss, NPWT parameters, and dressing change frequency were collected. 

 The main outcome was SSI and additional outcomes were wound dehiscence, seroma, hospital length of stay, and 30-day mortality. The risk of bias was assessed with the Cochrane Risk of Bias 2 tool for randomized trials (RoB 2) by two independent reviewers (M.O.F. and R.O.M.), with disagreements resolved by consensus^45^. Publication bias was explored via funnel plot inspection. 

 The certainty of evidence was assessed using the Grading of Recommendations Assessment, Development and Evaluation (GRADE) framework, following the *Cochrane Handbook* and the GRADE Working Group^5^. Five domains were evaluated: risk of bias, inconsistency, indirectness, imprecision, and publication bias. 

### Statistical analysis

 Analyses were conducted using Review Manager (v5.4, Cochrane Collaboration) and Python (v3.12.3; NumPy, SciPy, statsmodels). Risk ratios (RRs) with 95% confidence intervals (CIs) were calculated for dichotomous outcomes (SSI, dehiscence, seroma, mortality), and mean differences (MDs) for continuous outcomes (length of stay). Random-effects models with the DerSimonian–Laird (DL) method were applied. Heterogeneity was quantified using the I2 statistic, with I^2^≥50% indicating substantial heterogeneity. Sensitivity analyses were performed by sequential exclusion of individual studies. Subgroup analyses to explore heterogeneity in SSI outcomes were planned for wound classification (clean contaminated *versus* dirty) and NPWT pressure levels. Artificial intelligence tools were used exclusively for language editing and grammatical revision. The use of these tools did not influence the study methodology, results, or interpretation. 

## RESULTS

### Study selection and baseline characteristics

**Figure 1:**
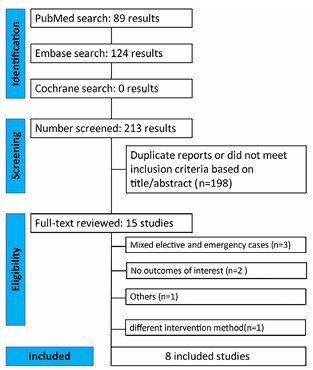
PRISMA flow diagram of study screening and selection.

 The initial search yielded 244 results. After removal of duplicates and ineligible studies, 15 studies remained and were fully reviewed based on the inclusion criteria. Of these, eight RCTs were included, comprising 1,377 patients^10,17,20,31,37,42,45,47^. The main reasons for exclusion were mixed-case studies, lack of relevant outcomes, and incorrect intervention. Figure 1 describes the detailed study selection process. 

 A total of 707 (51.3%) patients received NPWT, and 670 (48.7%) received conventional dressings. Study characteristics are reported in Table 1. The mean age and gender distribution were similar between studies. Two studies focused on peritonitis patients^42,45^. The remaining studies included all patients undergoing emergency laparotomy, with a majority of clean-contaminated and dirty wound classifications^36^. 

**Table 1:** Study characteristics.

Study	Type of study	Location, Period	Patients n (%) NPWT/Conv.	Age, years NPWT/Conv.	Female n (%) NPWT/Conv.	BMI kg/m^2^ NPWT/Conv.	Hypertension n (%) NPWT/Conv.	Diabetes n (%) NPWT/Conv.	Smoker n (%) NPWT/Conv.	Active malignancy n (%) NPWT/Conv.	Serum Albumin (g/L) NPWT/Conv.
Lozano-Balderas et al.^31^	RCT	Mexico, NA	25 (31)/27 (33)	32/30	6 (24)/7 (25.9)	25.90/26.70	NA	NA	16.0/22.2	NA	3.60/3.40
Garg et al.^17^	RCT	India, 2018–2020	25 (50)/25 (50)	46.76/41.96	13 (52)/9 (36)	NA	NA	NA	NA	NA	NA
Singh et al.^45^	RCT	India, 2018–2021	34 (52.3)/31 (47.7)	37/33	8 (23)/5 (16)	23.20/22.20	1 (3)/ 1 (3)	NA	13 (38)/ 9 (29)	NA	2.80/2.70
Costa et al.^10^	RCT	Portugal, 2022–2023	80 (65)/43 (35)	63.46/64.31	22 (27.6)/16 (37.2)	NA	NA	15 (27.4)/28 (22.7)	15 (18.7)/26 (21.1)	NA	NA
Philip et al.^37^	RCT	Malaysia, 2020–2022	47 (49)/49 (51)	NA	21 (44)/22 (44.9)	NA	NA	10 (21.3)/6 (12.2)	5 (10.6)/6 (12.2)	4 (8.5)/10 (20.4)	NA
Sahni et al.^42^	RCT	India, 2018–2020	40 (50)/40 (50)	40 (50)/40 (50)	40 (50)/40 (50)	40 (50)/40 (50)	NA	NA	NA	NA	5.38/5.35
Herczeg et al.^20^	RCT	Hungary, NA	45 (50)/45 (50)	60.96/67.67	8 (19)/11 (24)	26.64/27.38	NA	8 (15.1)/7 (15.2)	17 (37.8)/15 (33.3)	NA	36.03/34.53
SUNRRISE^47^	RCT	Multinational, 2018–2021	411 (49.9)/410 (40.1)	68.3/63.7	207 (50.4)/224 (54.6)	27.10/27.20	NA	40 (9.7)/40 (9.8)	95 (23.1)/70 (17.1)	85 (20.4)/76 (18.5)	3.39/3.46

BMI: body mass index; Conv.: conventional dressings; NA: not available data; NPWT: negative pressure wound therapies; RCT: randomized control trial.

 The negative pressure used to prepare the dressing for the intervention group ranged from 75 to 120 mmHg. Other surgical characteristics are reported in Table 2. 

**Table 2:** Surgical characteristics.

Study	Diagnosis	Negative pressure, mmHg NPWT/Conv.	CDC Clean n (%) NPWT/Conv.	CDC Clean-Contaminated n (%) NPWT/Conv.	CDC Contaminated n (%) NPWT/Conv.	CDC Dirty n (%) NPWT/Conv.	Dressing time frequency, days
Lozano-Balderas et al.^31^	Benign perforation, Stab wound, Acute diverticulitis, Strangulated hernia, Others	NA	0/0	0/0	48/33.3	52/66.7	2/2
Garg et al.^17^	NA	75/NA	NA	NA	NA	NA	3/4.28
Singh et al.^45^	Perforation	120/NA	0/0	0/0	0/0	100/100	4/1
Costa et al.^10^	Intestinal occlusion, Ischemic emergencies, Abdominal wall hernia, Abdominal trauma, Abdominal sepsis, Gastrointestinal bleeding	80–125/NA	19 (23.8)/10 (23.3)	19 (23.6)/16 (37.2)	24 (29.95)/5 (11.6)	18 (22.65)/12 (27.9)	7/2
Philip et al.^37^	Trauma, Benign obstruction, Benign perforation, Malignant perforation, Ischemia, Bleeding	80/NA	6 (12.8)/1 (2)	26 (55.3)/15 (51)	5 (10.6)/4 (8.2)	10 (21.3)/19 (37.5)	7/7
Sahni et al.^42^	Peritonitis	75/40	NA	NA	NA	NA	4/2
Herczeg et al.^20^	NA	70–120/0	1 (2.2)/0 (0)	3 (6.7)/7 (15.6)	26 (57.8)/21 (37.8)	15 (33.3)/17 (37.8)	5/5
SUNRRISE^47^	NA	80/28.5	98 (23.8)/99 (24.1)	175 (42.6)/176 (42.9)	81 (19.7)/79 (19.3)	57 (13.9)/56 (13.7)	7/3.46

NA: not available data; NPWT: negative pressure wound therapies; CDC: Centers for Disease Control and Prevention; Conv.: conventional dressings.

### Pooled analysis of all studies

 The main outcome (SSI) was assessed across all eight RCTs. Additional outcomes, including wound dehiscence, seroma formation, hospital length of stay, and 30-day mortality, were evaluated where data were available. 

 For SSI (eight studies, total of 1,377 patients)^10,17,20,31,37,42,45,47^, NPWT significantly reduced risk compared to conventional dressings (RR 0.43; 95%CI 0.26–0.73; p=0.002). However, substantial heterogeneity was observed (I^2^=76%; p<0.001), which revealed a consistent direction of effect favoring NPWT across studies, though the magnitude varied (Table 3). 

**Table 3:** Comparison of surgical site infection between negative pressure wound therapy and conventional dressings.

Study	NPWT events	NPWT total	Conv. events	Conv. total	Weight (%)	Risk ratio (M-H, Random, 95%CI)
Costa et al.^10^	8	80	13	43	13.8	0.33 (0.15–0.74)
Garg et al.^17^	3	25	8	25	9.8	0.38 (0.11–1.25)
Herczeg et al.^20^	10	45	20	45	15.6	0.50 (0.26–0.95)
Lozano-Balderas et al.^31^	0	25	15	56	3.0	0.07 (0.00–1.14)
Philip et al.^37^	2	47	7	49	7.5	0.30 (0.07–1.36)
Sahni et al.^42^	12	40	28	40	16.9	0.43 (0.26–0.72)
Singh et al.^45^	6	34	19	31	14.0	0.29 (0.13–0.63)
SUNRRISE^47^	112	394	108	394	19.5	1.04 (0.83–1.30)
Total	—	690	—	683	100.0	0.43 (0.26–0.73)

NPWT: negative pressure wound therapies; Conv.: conventional dressings; M-H: Mantel-Haenszel; CI: confidence interval.

 The total number of events in the NPWT group was 153, while in the conventional dressing group it was 218. The pooled analysis showed a significant reduction in the risk of surgical site infection with NPWT compared with conventional dressings (RR 0.43; 95%CI 0.26–0.73; z=3.14, p=0.002), although substantial heterogeneity was observed across studies (tau^2^ DerSimonian–Laird [DL]=0.35; ꭓ^2^=29.67; degrees of freedom [df]=7, p=0.0001; I^2^=76%). 

 Wound dehiscence was reported in five RCTs (n=231 NPWT; n=193 control)^10,16,17,37^. NPWT was associated with a statistically significant reduction in risk (RR 0.32; 95%CI 0.180.58; p=0.0002), with no evidence of heterogeneity (I^2^=0%; p=0.80), and with uniform effect sizes across studies (Table 4). 

**Table 4:** Comparison of negative pressure wound therapy with conventional dressings, for dehiscence.

Study	NPWT events	NPWT total	Conv. events	Conv. total	Weight (%)	Risk ratio (M-H, Random, 95%CI)
Costa et al.^10^	2	80	4	43	13.0	0.27 (0.05–1.41)
Garg et al.^17^	2	25	4	25	13.8	0.50 (0.10–2.49)
Herczeg et al.^20^	1	45	2	45	6.4	0.50 (0.05–5.32)
Philip et al.^37^	5	47	13	49	39.4	0.40 (0.15–1.04)
Singh et al.^45^	3	34	15	31	27.4	0.18 (0.06–0.57)
Total	—	231	—	193	100.0	0.32 (0.18–0.58)

NPWT: negative pressure wound therapies; Conv.: conventional dressings; M-H: Mantel-Haenszel; CI: confidence interval.

 The total number of events in the NPWT group was 13, while in the conventional dressing group it was 38. The pooled analysis showed a significant reduction in the risk of surgical site infection with NPWT compared with conventional dressings (RR 0.32; 95%CI 0.18–0.58; z=3.73, p=0.0002), with no evidence of heterogeneity across studies (tau^2^ [DL]=0.00; ꭓ^2^=1.64; degrees of freedom [df]=4, p=0.80; I^2^=0%). 

 Seroma formation was reported in three RCTs (n=152 NPWT; n=117 control)^10,17,37^. The point estimate suggested a benefit of NPWT (RR 0.59; 95%CI 0.34–1.01; p=0.05), with no heterogeneity (I2=0%) (Table 5). 

**Table 5:** Comparison of negative pressure wound therapy with conventional dressings, for seroma.

Study	NPWT Events	NPWT Total	Conv. events	Conv. total	Weight (%)	Risk ratio (M-H, Random, 95%CI)
Costa et al.^10^	5	80	4	43	18.3	0.67 (0.19–2.37)
Garg et al.^17^	1	25	2	25	5.3	0.50 (0.05–5.17)
Philip et al.^37^	11	47	20	49	76.4	0.57 (0.31–1.06)
Total	—	152	—	117	100.0	0.59 (0.34–1.01)

NPWT: negative pressure wound therapies; Conv.: conventional dressings; M-H: Mantel-Haenszel; CI: confidence interval.

 The total number of events in the NPWT group was 17, while in the conventional dressing group it was 26. The pooled analysis suggested a reduction in the risk of surgical site infection with NPWT compared with conventional dressings, although the result was of borderline statistical significance (z=1.94, p=0.05), with no evidence of heterogeneity across studies (tau^2^ [DL]=0.00; ꭓ^2^=0.07; degrees of freedom [df]=2, p=0.97; I^2^=0%). 

 Hospital length of stay was evaluated in six RCTs (n=199 NPWT; n=192 control)^17,20,37,42,45,47^. The pooled estimate indicated no significant difference between NPWT and conventional dressings (MD -0,39 days; 95%CI -1.15–0.36; p=0.9227), with low heterogeneity (I^2^=0%; p=0.9227). The forest plot shows CIs consistently overlapping with the null effect line (Table 6). 

**Table 6:** Comparison of negative pressure wound therapy with conventional dressings, for length of hospital stays.

Study	NPWT (n)	NPWT (mean)	NPWT (SD)	Control (n)	Control (mean)	Control (SD)	MD (95%CI)	Weight (%)
Philip et al.^37^	30	9.00	5.93	30	11.00	12.22	-2.00 (-6.86–2.86)	4.9
Singh et al.^45^	34	9.00	3.70	31	10.00	5.93	-1.00 (-3.43–1.43)	19.7
SUNRRISE^47^	30	8.00	5.93	30	9.00	6.30	-1.00 (-4.10–2.10)	12.1
Sahni et al.^42^	30	9.50	7.78	30	10.00	11.30	-0.50 (-5.41–4.41)	4.8
Garg et al.^17^	30	8.20	2.34	30	8.21	3.37	-0.01 (-1.48–1.46)	53.9
Herczeg et al.^20^	45	13.42	15.35	45	12.31	7.73	1.11 (-3.91–6.13)	4.6
Random effects model	199	—	—	196	—	—	-0.39 (-1.15–0.36)	100.0

NPWT: negative pressure wound therapies; SD: standard deviation; MD: mean difference; CI: confidence interval.

Heterogeneity: I^2^=0.0%/Tau^2^=0/p=0.9227.

 For 30-day mortality, data were available from four RCTs (n=570 NPWT; n=529 control)^10,20,45,47^. No significant difference was observed between NPWT and conventional dressings (RR 0.96; 95%CI 0.55–1.68; p=0.88), with no heterogeneity (I^2^=0%) (Table 7). 

**Table 7:** Comparison of negative pressure wound therapy with conventional dressings, for 30-day mortality.

Study	NPWT events	NPWT total	Conv. events	Conv. total	Weight (%)	Risk ratio (M-H, Random, 95%CI)
Costa et al.^10^	7	80	2	43	13.4	1.88 (0.41–8.66)
Herczeget al.^20^	7	45	5	45	27.2	1.40 (0.48–4.08)
Singh et al.^45^	2	34	3	31	10.5	0.61 (0.11–3.40)
SUNRRISE^47^	10	411	14	410	48.8	0.71 (0.32–1.59)
Total	—	570	—	529	100.0	0.96 (0.55–1.68)

NPWT: negative pressure wound therapies; Conv.: conventional dressings; M-H: Mantel-Haenszel; CI: confidence interval.

### Subgroup and sensitivity analyses

 Subgroup analyses in SSI outcomes were planned for wound classification (clean contaminated *versus* dirty) and NPWT pressure levels, but were constrained by inconsistent reporting across studies. Leave-one-out sensitivity analyses confirmed the stability of SSI and dehiscence findings, with no single study disproportionately influencing the pooled estimates. 

### Quality assessment

 The overall risk of bias across the included randomized trials was predominantly rated as “some concerns”^10,17,20,31,37,45^, with only one study achieving a low risk of bias in all domains^46^. The most frequent sources of potential bias arose from deviations from the intended interventions and the randomization process, reflecting occasional limitations in allocation concealment and blinding^10,17,20,31,37,45^. While missing outcome data and selective reporting were generally low risk, certain trials demonstrated some concerns in outcome measurement, primarily due to the lack of assessor blinding for subjective endpoints^10,19,20,31^. One trial was judged to be at high risk of bias, driven by deficiencies in the randomization process and deviations from intended interventions^9^. 

 Randomization was adequately reported in Garg et al.^17^, Singh et al.^45^, and SUNRRISE Trial Study Group^47^ (low risk), while four studies lacked sufficient details^10,20,31,37^. Costa et al.^10^ was rated as high risk for predictable allocation. Lack of blinding led to some concerns in three trials, whereas adherence was adequate in the others. Attrition was <10% across all studies, with intention-to-treat analyses ensuring low risk. Outcome assessment was mostly objective, though four trials had unblinded assessors. No selective reporting bias was detected. Overall, six studies presented some concerns, and Costa et al.^10^ was high risk; SUNRRISE Trial Study Group^47^ showed low overall risk. 

 In the GRADE evaluation, primary outcomes included SSI, wound dehiscence, seroma formation, length of hospital stay, and 30-day mortality. Risk of bias and inconsistency did not justify downgrading. Evidence was directly applicable to the target population. Imprecision resulted in moderate certainty for seroma, hospital stay, and mortality, while SSI and dehiscence were supported by high-certainty evidence. No publication bias was identified, as all trials were registered and published in peer-reviewed journals. In funnel plot analysis, studies occupied a symmetrical distribution according to weight and converged towards the pooled effect as weight increased (Table 7). 

## DISCUSSION

 The evidence presented in this systematic review and metaanalysis of eight RCTs supports potential benefits of NPWT compared to conventional dressing methods in emergency laparotomy. 

 The main findings from the pooled analysis were as follows:

1. NPWT significantly reduced the risk of SSI;
2. NPWT was associated with a statistically significant reduction in dehiscence; and
3. No significant effects were observed on seroma formation, hospital length of stay, or 30-day mortality.

This study’s results are consistent with previous cohort investigations, which demonstrated a statistically significant decrease in SSI among patients undergoing emergency laparotomy^43,44,48^. A meta-analysis by Groenen et al.^19^ synthesizing 57 RCTs with 13,744 patients also showed that incisional NPWT significantly reduced SSI across various surgical specialties with a relative risk of 0.67. On the other hand, a cohort study analyzing 65,803 patients with completely closed incisions, of whom 387 received NPWT, concluded there was no significant difference in the rate of SSI between NPWT and standard dressings (13.4 *vs.* 11.9%), raising important questions regarding its efficacy in specific surgical contexts^37^. 

 Previous systematic reviews and meta-analyses have demonstrated a significant reduction in wound dehiscence with NPWT, particularly in contaminated and high-risk incisions^28,30,41^. Corroborating these reports, the present metaanalysis showed a 68% relative risk reduction in dehiscence with NPWT. Mechanistically, NPWT could confer this benefit by stabilizing the incision, redistributing lateral tension, and enhancing perfusion and clearance of inflammatory mediators^41,50^. 

 The analysis of postoperative seroma formation, including three RCTs, showed no significant reduction in postoperative seroma formation, a finding consistent with a previous meta-analysis^29^. The effect of NPWT on hospital length of stay was also not significant in our study, aligning with the conclusions of Lakhani et al.^28^ and Almansa-Saura et al.^1^ In addition, we found no significant effect on 30-day mortality, analyzed in three RCTs. No other meta-analysis has been identified as assessing this outcome due to limited and heterogeneous reporting of these outcomes in included studies. 

 This meta-analysis has several limitations. First, the main outcome of SSI showed substantial heterogeneity, potentially driven by differences in wound contamination levels, NPWT protocols (e.g., pressure and duration), and patient comorbidities across studies, as assessed using GRADE criteria (low certainty due to inconsistency)^5^. Although sensitivity analyses confirmed that no single study was driving heterogeneity, subgroup explorations were limited by incomplete reporting of key variables like CDC wound class, precluding definitive attribution of sources. Second, additional outcomes relied on data from few RCTs, yielding wide CIs and imprecise estimates (very low certainty). Third, most included trials were small and single-center, except the SUNRRISE Trial Study Group^47^, raising concerns about generalizability to diverse populations and settings. Fourth, risk of bias concerns were present in several domains (e.g., blinding), though low in missing data and reporting. Publication bias appeared minimal via funnel plots, but small-study effects cannot be ruled out. Finally, cost-effectiveness and long-term outcomes (beyond 30 days) were not evaluated, limiting clinical implications. 

 Considering the substantial heterogeneity, future studies should aim to stratify patient populations more effectively and standardize NPWT protocols to enhance the robustness of findings. In addition, the results suggest that while NPWT may reduce SSIs and dehiscence rates, its impact on 30-day mortality, seroma formation, and hospital length of stay in emergency laparotomies is less clear. Further research is needed to investigate these outcomes further and clarify if specific patient characteristics are associated with the efficacy of NPWT. Recent large RCTs, such as the SUNRRISE Trial Study Group^47^, have reported no benefit, which could be partially attributed to the need for careful patient selection. 

## CONCLUSIONS

 This original systematic review and meta-analysis of RCTs demonstrated the efficacy of NPWT in reducing the incidence of SSIs and wound dehiscence among patients undergoing emergency laparotomy when compared to conventional dressings. 

## Data Availability

The datasets generated and/or analyzed during the current study are available from the corresponding author upon reasonable request.

## References

[1] Almansa-Saura S, Lopez-Lopez V, Eshmuminov D, Schneider M, Castellanos-Escrig G, Rodriguez-Valiente M (2021). Prophylactic use of negative pressure therapy in general abdominal surgery: a systematic review and meta-analysis. Surg Infect (Larchmet).

[2] Andrade EG, Guerra JJ, Punch L (2020). A multi-modal approach to closing exploratory laparotomies including high-risk wounds. Cureus.

[3] Atkins BZ, Wooten MK, Kistler J, Hurley K, Hughes GC, Wolfe WG (2009). Does negative pressure wound therapy have a role in preventing poststernotomy wound complications?. Surg Innov.

[4] Badia JM, Casey AL, Petrosillo N, Hudson PM, Mitchell SA, Crosby C (2017). Impact of surgical site infection on healthcare costs and patient outcomes: a systematic review in six European countries. J Hosp Infect.

[5] Balshem H, Helfand M, Schünemann HJ, Oxman A, Kunz R, Brozek J (2011). GRADE guidelines: 3. Rating the quality of evidence. J Clin Epidemiol.

[6] Broex ECJ, van Asselt ADI, Bruggeman CA, van Tiel FH (2009). Surgical site infections: how high are the costs?. J Hosp Infect.

[7] Calò PG, Catena F, Corsaro D, Costantini L, Falez F, Moretti B (2023). Guidelines for improvement of the procedural aspects of devices and surgical instruments in the operating theatre. Front Surg.

[8] Chambers LM, Morton M, Lampert E, Yao M, Debernardo R, Rose P (2020). Use of prophylactic closed incision negative pressure therapy is associated with reduced surgical site infections in gynecologic oncology patients undergoing laparotomy. Am J Obstet Gynecol.

[9] Chung JNC, Ali O, Hawthornthwaite E, Watkinson T, Blyth U, McKigney N (2021). Closed incision negative pressure wound therapy is associated with reduced surgical site infection after emergency laparotomy: a propensity matched cohort analysis. Surgery.

[10] Costa MJ, Martins MF, Lages RR, Gonçalves AL, Armas IS, Almeida JI (2024). Optimizing closed incision negative pressure wound therapy in emergency laparotomy (OPTIWOUND): a multi-arm randomized prospective trial. Surg Gastroenterol Oncol.

[11] Costabella F, Patel KB, Adepoju AV, Singh P, Mahmoud HAH, Zafar A (2023). Healthcare cost and outcomes associated with surgical site infection and patient outcomes in low- and middle-income countries. Cureus.

[12] de-Aguilar-Nascimento JE, Salomão AB, Waitzberg DL, Dock-Nascimento DB, Corrêa MITD, Campos ACL (2017). ACERTO guidelines of perioperative nutritional interventions in elective general surgery. Rev Col Bras Cir.

[13] Lissovoy G, Fraeman K, Hutchins V, Murphy D, Song D, Vaughn BB (2009). Surgical site infection: incidence and impact on hospital utilization and treatment costs. Am J Infect Control.

[14] DeCarbo WT, Hyer CF (2010). Negative-pressure wound therapy applied to high-risk surgical incisions. J Foot Ankle Surg.

[15] Freitas ACT, Ferraz AAB, Barchi LC, Boin IFSF (2023). Antibiotic prophylaxis for abdominal surgery: when to recommend? Brazilian College of Digestive Surgery position paper. Arq Bras Cir Dig.

[16] Garale MN, Rewatkar AK, Moktali AV, Dalvi A (2025). Incidence and risk factors for surgical site infections following emergency laparotomies: a prospective observational study. Cureus.

[17] Garg A, Jayant S, Gupta AK, Bansal LK, Wani A, Chaudhary P (2021). Comparison of closed incision negative pressure wound therapy with conventional dressing for reducing wound complications in emergency laparotomy. Pol J Surg.

[18] Gombert A, Babilon M, Barbati ME, Keszei A, von Trotha K, Jalaie H (2018). Closed incision negative pressure therapy reduces surgical site infections in vascular surgery: a prospective randomised trial (AIMS Trial). Eur J Vasc Endovasc Surg.

[19] Groenen H, Jalalzadeh H, Buis DR, Dreissen YEM, Goosen JHM, Griekspoor M (2023). Incisional negative pressure wound therapy for the prevention of surgical site infection: an up-to-date meta-analysis and trial sequential analysis. EClinicalMedicine.

[20] Herczeg A, Szijártó A, Fülöp A, Varga K, Marton J, Lóderer Z (2025). Prophylactic negative pressure wound therapy reduces superficial surgical site infection risk of emergency surgery patients: results of a multicenter randomised prospective clinical trial. Int Wound J.

[21] Higgins JPT, Cochrane Collaboration (2019). Cochrane handbook for systematic reviews of interventions.

[22] Hou Y, Collinsworth A, Hasa F, Griffin L (2022). Incidence and impact of surgical site infections on length of stay and cost of care for patients undergoing open procedures. Surg Open Sci.

[23] Imtiaz H, Ali C, Noordeen H, Anwar H (2024). PICO™ (Closed-Incision Negative-Pressure Wound Therapy) dressing use as postoperative prophylaxis for preventing surgical site infections in spinal surgery: a retrospective single-centre study. Cureus.

[24] Jeffery S, Leaper D, Armstrong D, Lantis J (2018). Using negative pressure wound therapy to prevent surgical site infection. J Wound Care.

[25] Jenks PJ, Laurent M, McQuarry S, Watkins R (2014). Clinical and economic burden of surgical site infection (SSI) and predicted financial consequences of elimination of SSI from an English hospital. J Hosp Infect.

[26] Kenig J, Richter P, Żurawska S, Lasek A, Zbierska K (2012). Risk factors for wound dehiscence after laparotomy – clinical control trial. Pol Przegl Chir.

[27] Kugler NW, Carver TW, Paul JS (2016). Negative pressure therapy is effective in abdominal incision closure. J Surg Res.

[28] Lakhani A, Jamel W, Riddiough GE, Cabalag CS, Stevens S, Liu DS (2022). Prophylactic negative pressure wound dressings reduces wound complications following emergency laparotomies: a systematic review and meta-analysis. Surgery.

[29] Liberati A, Altman DG, Tetzlaff J, Mulrow C, Gøtzsche P, Ioannidis J (2009). The PRISMA statement for reporting systematic reviews and meta-analyses of studies that evaluate health care interventions: explanation and elaboration. PLoS Med.

[30] Liu DS, Cheng C, Islam R, Tacey M, Sidhu A, Lam D (2021). Prophylactic negative-pressure dressings reduce wound complications and resource burden after emergency laparotomies. J Surg Res.

[31] Lozano-Balderas G, Ruiz-Velasco-Santacruz A, Díaz-Elizondo JA, Gómez-Navarro JA, Flores-Villalba E (2017). Surgical site infection rate drops to 0% using a vacuum-assisted closure in contaminated/dirty infected laparotomy wounds. Am Surg.

[32] Lynam S, Mark KS, Temkin SM (2016). Primary placement of incisional negative pressure wound therapy at time of laparotomy for gynecologic malignancies. Int J Gynecol Cancer.

[33] Meyer J, Roos E, Davies RJ, Buchs NC, Ris F, Toso C (2023). Does prophylactic negative-pressure wound therapy prevent surgical site infection after laparotomy? A Systematic Review and Meta-analysis of Randomized Controlled trials. World J Surg.

[34] Modi J, Patel Y, Trivedi M, Bochiya G (2023). An abdominal wound dehiscence of emergency explorative laparotomy and their management at tertiary care centre: an observational study. Int Surg J.

[35] Nakatsutsumi K, Endo A, Asano H, Shinohara S, Kurosaki R, Kawashima S (2022). Prophylactic effect of negative-pressure wound therapy and delayed sutures against incisional surgical site infection after emergency laparotomy for colorectal perforation: a multicenter retrospective cohort study. Ann Gastroenterol Surg.

[36] Normandin S, Safran T, Winocour S, Chu C, Vorstenbosch J, Murphy A (2021). Negative pressure wound therapy: mechanism of action and clinical applications. Semin Plast Surg.

[37] Philip EF, Rajandram R, Zuber M, Khong TL, Roslani AC (2024). Prophylactic PICO dressing shortens wound dressing requirements post emergency laparotomy (EL-PICO trial). World J Emerg Surg.

[38] Rafaqat W, Proaño Zamudio JA, Abiad M, Lagazzi E, Argandykov D, Luckhurst C (2024). Negative pressure wound therapy for emergency laparotomy incisions: a national database propensity matched study. Am J Surg.

[39] Reddix RN, Leng XI, Woodall J, Jackson B, Dedmond B, Webb LX (2010). The effect of incisional negative pressure therapy on wound complications after acetabular fracture surgery. J Surg Orthop Adv.

[40] Roos E, Douissard J, Abbassi Z, Buchs N, Toso N, Ris F (2021). Prophylactic negative-pressure wound therapy for prevention of surgical site infection in abdominal surgery: a nationwide cross-sectional survey. Updates Surg.

[41] Sahebally SM, McKevitt K, Stephens I, Fitzpatrick F, Deasy J, Burke J (2018). Negative pressure wound therapy for closed laparotomy incisions in general and colorectal surgery: a systematic review and meta-analysis. JAMA Surg.

[42] Sahni K, Hosamani S, Ghuliani D, Baisoya S (2024). Evaluation of negative pressure dressings for closed surgical incisions in decreasing surgical site infections after emergency laparotomy: a randomized controlled study. Cureus.

[43] Sato Y, Sunami E, Hirano K, Takahashi M, Kosugi SI (2022). Efficacy of prophylactic negative-pressure wound therapy with delayed primary closure for contaminated abdominal wounds. Surg Res Pract.

[44] Schurtz E, Differding J, Jacobson E, Maki C, Ahmeti M (2018). Evaluation of negative pressure wound therapy to closed laparotomy incisions in acute care surgery. Am J Surg.

[45] Singh H, Avudaiappan M, Kharel J, Irrinki S, Kumar H, Savlania A (2023). Negative pressure wound therapy versus standard care for incisional laparotomy subcutaneous wounds in gastrointestinal perforations: a randomized controlled study. Surgery.

[46] Sterne JAC, Savović J, Page MJ, Elbers RG, Blencowe NS, Boutron I (2019). RoB 2: a revised tool for assessing risk of bias in randomised trials. BMJ.

[47] Atherton K, Brown J, Clouston H, Coe P, Duarte R, SUNRRISE Trial Study Group (2025). Negative pressure dressings to prevent surgical site infection after emergency laparotomy: the SUNRRISE randomized clinical trial. JAMA.

[48] Tsukazaki Y, Enomoto H, Takeuchi N, Ushigome T, Suwa K, Okamoto T (2024). Incisional negative pressure wound therapy for wounds in patients with lower intestinal perforations. J Anus Rectum Colon.

[49] Yu L, Kronen RJ, Simon LE, Stoll CRT, Colditz GA, Tuuli MG (2018). Prophylactic negative-pressure wound therapy after cesarean is associated with reduced risk of surgical site infection: a systematic review and meta-analysis. Am J Obstet Gynecol.

[50] Zaidi A, El-Masry S (2017). Closed-incision negative-pressure therapy in high-risk general surgery patients following laparotomy: a retrospective study. Colorectal Dis.

[51] Zaidi MY, Nussbaum DP, Hsu SD, Strickler J, Uronis H (2023). Hepatic artery infusion for unresectable colorectal cancer liver metastases. Surgery.

[52] Zhu Y, Dai L, Luo B, Zhang L (2023). Meta-analysis of prophylactic negative pressure wound therapy for surgical site infections in caesarean section surgery. Wideochir Inne Tech Maloinwazyjne.

[53] Zimlichman E, Henderson D, Tamir O, Franz C, Song P, Yamin CK (2013). Health care-associated infections: a meta-analysis of costs and financial impact on the US health care system. JAMA Intern Med.

